# What gives rise to clinician gut feeling, its influence on management decisions and its prognostic value for children with RTI in primary care: a prospective cohort study

**DOI:** 10.1186/s12875-018-0716-7

**Published:** 2018-02-05

**Authors:** Sophie Turnbull, Patricia J. Lucas, Niamh M. Redmond, Hannah Christensen, Hannah Thornton, Christie Cabral, Peter S. Blair, Brendan C. Delaney, Matthew Thompson, Paul Little, Tim J. Peters, Alastair D. Hay

**Affiliations:** 1 Centre for Academic Primary Care, Population Health Sciences, Bristol Medical School, University of Bristol, Canynge Hall, 39 Whatley Road, Bristol, BS8 2PS UK; 2 School for Policy Studies, University of Bristol, 8 Priory Road, Bristol, BS8 1TZ UK; 3 National Institute for Health Research Collaborations for Leadership in Applied Health Research and Care West (NIHR CLAHRC West), University Hospitals Bristol NHS Foundation Trust, 9th Floor, Whitefriars, Lewins Mead, Bristol, BS1 2NT UK; 4 Population Health Sciences, Bristol Medical School, University of Bristol, Oakfield House, Oakfield Grove, Bristol, BS8 2BN UK; 5 Population Health Sciences, Bristol Medical School, University of Bristol, Level D, St Michael’s Hospital, Southwell St, Bristol, BS2 8EG UK; 6 Department of Surgery and Cancer, Imperial College London, St Mary’s Hospital, W2 1NY, London, UK; 7 Department of Family Medicine, University of Washington, Seattle, WA 98195-4696 USA; 8 Primary Care and Population Sciences Unit, University of Southampton, Aldermoor Health Centre, Aldermoor Close, Southampton, SO16 5ST UK; 9 School of Clinical Sciences, University of Bristol, 69 St Michael’s Hill, Bristol, BS2 8DZ UK; 10 Centre for Academic Primary Care, School of Social and Community Medicine, University of Bristol, Bristol, BS8 2PS UK

**Keywords:** Child, Respiratory tract infections, Primary health care, Prognosis, Emotions, Decision making, Paediatric, Cough

## Abstract

**Background:**

The objectives were to identify 1) the clinician and child characteristics associated with; 2) clinical management decisions following from, and; 3) the prognostic value of; a clinician’s ‘gut feeling something is wrong’ for children presenting to primary care with acute cough and respiratory tract infection (RTI).

**Methods:**

Multicentre prospective cohort study where 518 primary care clinicians across 244 general practices in England assessed 8394 children aged ≥3 months and < 16 years for acute cough and RTI. The main outcome measures were: Self-reported clinician ‘gut feeling’; clinician management decisions (antibiotic prescribing, referral for acute admission); and child’s prognosis (reconsultation with evidence of illness deterioration, hospital admission in the 30 days following recruitment).

**Results:**

Clinician years since qualification, parent reported symptoms (illness severity score ≥ 7/10, severe fever < 24 h, low energy, shortness of breath) and clinical examination findings (crackles/ crepitations on chest auscultation, recession, pallor, bronchial breathing, wheeze, temperature ≥ 37.8 °C, tachypnoea and inflamed pharynx) independently contributed towards a clinician ‘gut feeling that something was wrong’. ‘Gut feeling’ was independently associated with increased antibiotic prescribing and referral for secondary care assessment. After adjustment for other associated factors, gut feeling was not associated with reconsultations or hospital admissions.

**Conclusions:**

Clinicians were more likely to report a gut feeling something is wrong, when they were more experienced or when children were more unwell. Gut feeling is independently and strongly associated with antibiotic prescribing and referral to secondary care, but not with two indicators of poor child health.

**Electronic supplementary material:**

The online version of this article (10.1186/s12875-018-0716-7) contains supplementary material, which is available to authorized users.

## Background

Acute cough with respiratory tract infection (RTI) in children is the most common problem managed by health services internationally [1, 2]. While the majority of childhood RTIs are self-limiting, a small number result in serious illness and hospitalisation [3]. Clinicians report that uncertainty regarding both the diagnosis and prognosis of some children with RTIs are important drivers of antibiotic prescribing, [4–6] contributing to over-prescribing [7] and antimicrobial resistance [8]. Knowing the prognosis of children presenting to primary care with acute RTIs can be challenging as clinicians cannot always be certain where in the illness trajectory the child has presented, and whether red flag symptoms and signs are absent or are yet to develop. In cases where there is uncertainty, clinical intuition or ‘gut feeling’ is thought to play a part in management decisions.

The current literature offers two definitions of clinician gut feeling. Both propose it arises when the clinician feels uncertain or has a low ‘feeling of rightness’, [9, 10] and both have been shown to influence management decisions [11]. In some studies, a comparison has been made between a sense of reassurance versus a feeling that ‘something is wrong’ which defines a broad set of cases where clinicians are worried [11]. In others, authors define a gut feeling as a sense of dissonance between intuitive and analytic reasoning; a gut feeling something is wrong despite a lack of clinical markers [9]. The latter definition draws on theories of clinical reasoning distinguishing between “intuitive” processing, which is rapid and unconscious and “analytic” processing that involves slow deliberative reasoning [12–14].

In primary care, gut feeling has been described as an incorporation of clinician knowledge, experience and information about the patient [9]. In the case of childhood RTI, clinicians report using immediately apparent symptoms and signs (the child’s energy, pallor and breathing) to distinguish severe from non-severe cases, using pattern recognition and drawing on past experience [5]. A recent study in children with any acute illness, found that: general appearance, breathing pattern, weight loss, history of convulsions and parental concern that the illness was different from any previously experienced predicted the feeling something was wrong when clinical impression was of non-serious illness [15]. This evidence goes some way to characterising the factors influencing clinical gut feeling, but does not identify the specific clinical features associated with particular illnesses, or provide objective assessments of illness severity.

Despite qualitative studies indicating the importance of clinician intuition for primary care doctors and in nursing, [16] there is a paucity of quantitative evidence regarding the prognostic value of gut feelings and whether primary care clinicians utilise them in their decision making. A systematic review suggested that gut feeling in itself should be viewed as a ‘diagnostic red flag’ and that it had greater diagnostic value than the majority of illness specific symptoms and signs [17]. Another study indicated that in situations where there was uncertainty after clinical assessment, gut feeling was highly predictive of serious infective illness [15].

We used a large prospective cohort study of children presenting to primary care with acute cough and RTI to address three objectives regarding ‘gut feeling that something is wrong’ (from here on ‘gut feeling’): (i) to describe the clinician and child characteristics that drive clinician to have a gut feeling that something is wrong; (ii) to investigate if gut feeling influences management decisions; and (iii) to evaluate the prognostic value of gut feeling in relation to primary care reconsultations with evidence of illness deterioration, and hospital admissions.

## Methods

### Design and setting

The ‘TARGET’ study [18] was a multicentre, prospective cohort study that recruited children presenting to general practices with acute cough and RTI, between July 2011 and May 2013. Practices were recruited and trained by four University hubs (Bristol, London, Oxford and Southampton). The primary aim of the study was to develop a clinical rule that could help clinicians improve their use of antibiotics by using baseline clinical characteristics to predict the children that would be hospitalised in the next 30 days [3]. Here we present findings from secondary analysis of this dataset, which are reported following the Strengthening the Reporting of Observational Studies in Epidemiology (STROBE) recommendations [19].

### Participants

Clinicians (General Practitioners: GPs and ‘prescribing’ Nurse Practitioners: NPs), were eligible to recruit to the study if they were working in participating primary care practices and reported that they prescribed antibiotics in ≤30% children with RTIs (to reduce the possibility of confounding by indication). Clinicians were asked to specify a priori their preferred recruitment strategy (for example, consecutive or first eligible on the day). Children were eligible if aged ≥3 months to < 16 years and presenting with an acute (≤28 days) cough and other symptoms of an RTI. Children with an infected exacerbation of asthma and those severely unwell (for example, requiring same day hospital assessment or admission) were included. Children were excluded if: presenting with a non-infective exacerbation of asthma; immuno-compromised; previously recruited or recently participated in other research; or temporarily registered at the GP practice.

### Data collection

#### Clinician characteristics

Clinician characteristics included the clinician type (GP or NP), number of years qualified and evidence of further medical/ health qualifications (Diploma in Child Health (DCH) or Membership of the Royal College of General Practitioners (MRCGP)).

#### Child characteristics

**Fig. 1:**
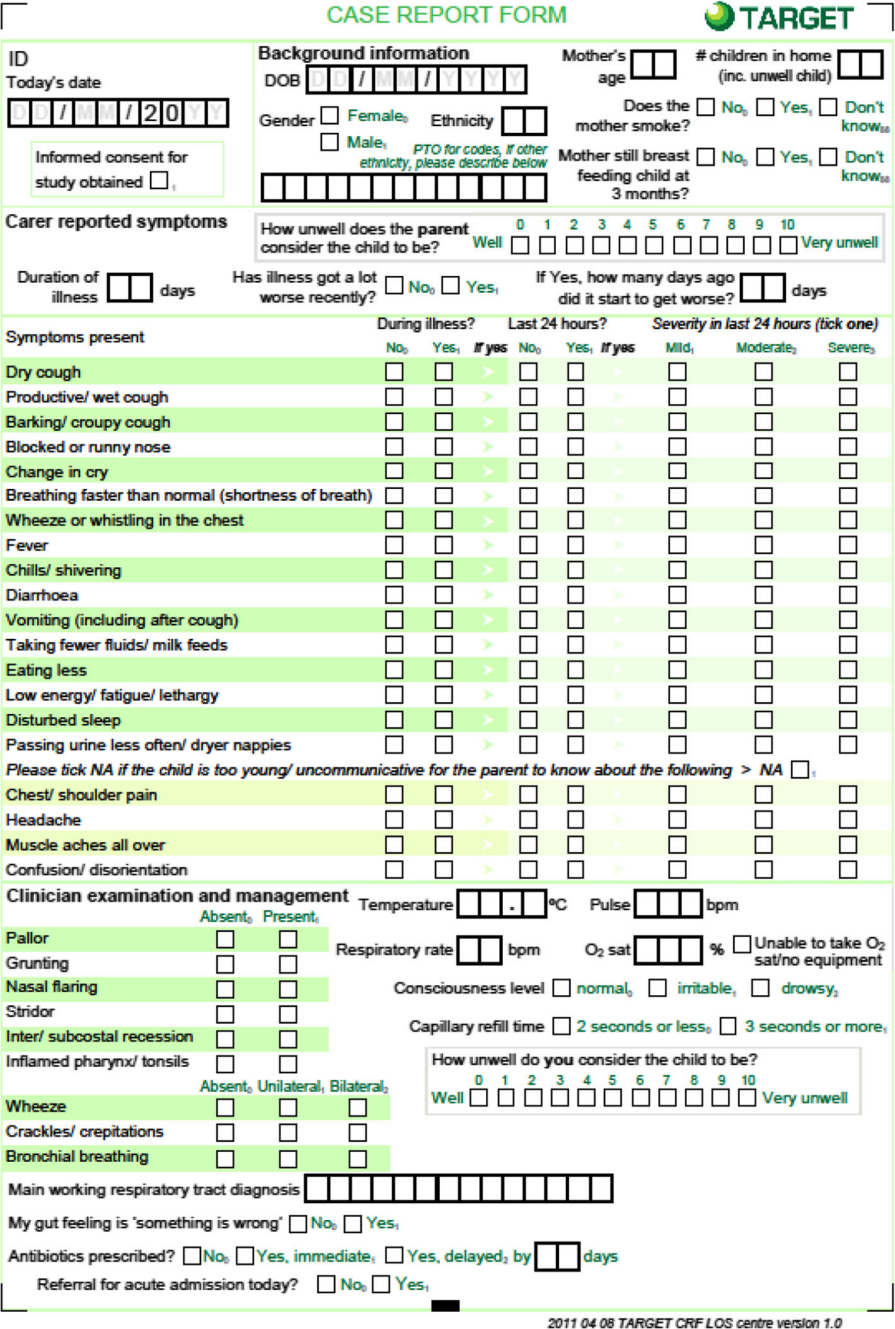
Case report form

At the baseline consultation the recruiting clinician used a structured case report form (CRF) to record socio-demographic information, parent-reported symptoms (including severity of symptoms in the past 24 h) and physical examination findings. A parent and clinician reported illness severity score was collected, scored 0-10 (see Fig. 1. Case report form). Information about the child’s past medical history was collected from the primary care medical record. The 2010 Indices of Multiple Deprivation (IMD) provided a measure of neighbourhood deprivation linked to the child’s home postcode [20].

#### Gut feeling

At the end of their clinical evaluation, the clinician was also asked to respond ‘yes’ or ‘no’ to the statement “my gut feeling is ‘something is wrong’” with the child. A definition was provided in the clinician instruction booklet that gut feeling in this context “represents clinicians gut feeling that the child’s illness may be more serious than is superficially apparent”. This question was located at the end of the CRF, following the socio-demographic, symptoms and physical examination findings, but prior to the reporting of management decisions.

#### Management decisions

Clinicians were asked to record if the child was referred for a secondary care assessment and if they prescribed antibiotics.

#### Prognostic value

Two indicators of poor child health were collected from the primary care medical record by trained notes reviewers at least 3 months after study entry (to allow sufficient time for the hospital discharge information to reach the medical record): primary care re-consultations for the same RTI with evidence of symptom deterioration; and hospital admissions for RTI in the 30 days following recruitment (using hospital discharge summaries). The 30 days threshold for hospitalisation was selected for two reasons. First, the rationale for the primary aim of the TARGET study [3] (from which the ‘gut feeling’ paper derives its data) was that clinicians report they prescribe antibiotics ‘just in case’ children are subsequently admitted to hospital with their RTI, and we sought to use an inclusive definition. Second, our previous [21] and more recent [22] research has shown the symptoms of acute cough and RTI take 25 days for most (90%) of children to resolve. A hospitalisation outcome with a ≤ 7 day cut off was also used as a sensitivity analysis to ensure that there was not a temporal association between the prognostic value of gut feeling and the hospitalisation outcome. We calculated the Kappa statistic to assess inter-rater reliability of the two reviewers that independently assessed the primary care medical record for these two health outcomes.

### Statistical analysis

#### Treatment of variables

Parent-reported symptom severity (mild, moderate, severe) in the 24 h prior to consultation was transformed into a binary outcome with a threshold dependent on overall prevalence: to either ‘severe/not severe’ (where > 5% of children reported as being severe or ‘moderate or severe/not moderate or severe’ (where < 5% of children reported being severe). This pragmatic cut-off was chosen prior to analysis to avoid severity variables with very low prevalence. We used accepted clinical cut offs (temperature > 37.8 °C, [23] capillary refill time (CRT) ≥3 s, [24, 25] oxygen saturation ≤ 95% [26]) or 25th percentile or 75th percentile thresholds to dichotomise continuous data, and we used age-adjusted thresholds for heart and respiratory rates [27]. Given the large number of variables being tested, continuous outcomes were initially categorised to help interpret and contextualise the final model, a sensitivity analysis was then conducted to assess the distribution of these continuous variables if included in the model.

#### Determinants of gut feeling

We first used univariable multilevel random effects logistic regression (accounting for between-clinician differences) to assess associations between clinician profile and children’s baseline characteristics, with gut feeling. Modelling was conducted using a multivariable multilevel random effects logistic regression model (accounting for clustering at the clinician level) and variable selection using the backward stepwise method with a 5% model entry threshold. For the ‘determinant’ analyses, the outcome variable was the clinician’s gut feeling and (given the large number of variables investigated) model retention was set at the 1% level. A more liberal threshold was employed for the univariable stage than for the multivariable stage because the former was used as a selection filter for the latter and we did not want to miss any potential associations of interest, whereas for the final selection of variables it was necessary to be more conservative given both the number of variables considered and the large sample size (which could potentially result in small effects being detected statistically). Global clinician illness severity score was not included in this analysis as it was suspected that there was overlap in what this and gut feeling variables represented to the clinician. Considering prior literature indicating prognostic value of gut feeling in the absence of clinical signs of severe illness, we undertook a sensitivity analysis to explore the contribution of high illness severity score to the model.

Evidence that clustering by clinician was important in the multivariable model was explored using the Likelihood-ratio test (LRT). The accuracy of the resulting model was represented by the Area Under the Receiver Operating Characteristic (AUROC) curve. Multiple imputation was employed as a sensitivity analysis to check whether the model was robust [28].

#### Management decisions and prognostic value

Univariable and multivariable multilevel logistic regression modelling was employed, using backward stepwise selection (accounting for between-clinician differences), as well as variables associated with each of those outcomes retaining variables with a *p*-value of < 0.05. Two models were used to investigate the associations between clinician gut feeling and the decision to: 1) prescribe antibiotics, and 2) refer the child to hospital for acute admission. Two further models were used to investigate the associations between gut feeling and: 1) re-consultation for the same RTI with evidence of deterioration, and; 2) hospitalisation for a RTI in the 30 days following recruitment. A further multivariable model was used in the sensitivity analysis exploring the association between gut feeling and the hospitalisation outcome with a ≤ 7 day cut off. To ensure the prognostic value of gut feeling did not differ with definition of gut feeling, a univariable analysis was conducted to explore the association between hospitalisation and the more focused definition described by a previous study: gut feeling when clinical impression was of non-serious illness [15]. Due to the exploratory nature of these analyses, and to ensure we did not miss any potential influences, the more liberal threshold was employed for the multivariable analysis. LRT was used to explore the influence of clustering by clinician in the multivariate models. Data were analysed using STATA version 13.1.

## Results

### Clinicians and children

Of the 842 clinicians agreeing to participate, 519 (62%) located at 247 general practices recruited at least one child (median 5, inter-quartile range (IQR) 2-15 children) into the study. Of the clinicians who recruited at least one child who was retained in the study, data were available about clinician type for 502 (97%) clinicians; of these 77 (15%) were NPs and 425 (85%) were GPs. Data on years since qualification were available for 498/518 (96%) of the clinicians: median of 20 years (range 1-44, IQR 13-26 years). Of the 519 clinicians, 85 (16%) reported having at least one additional qualification (DCH or MRCGP).

**Fig. 2:**
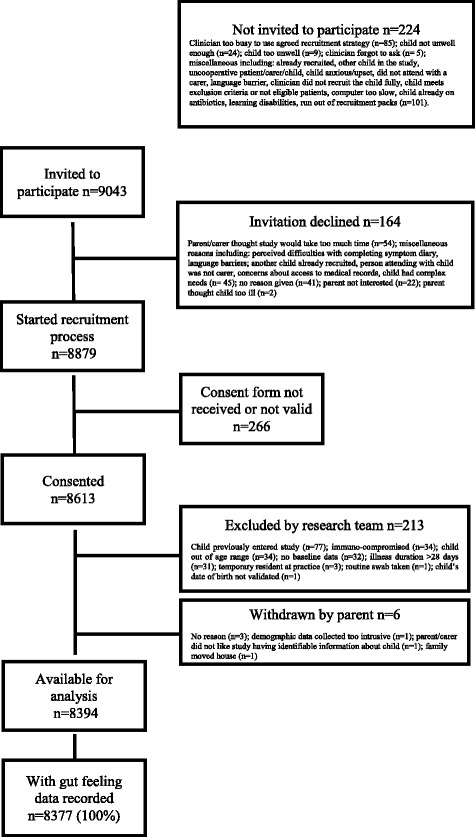
Flow of participants through the study

A total of 8613 children were recruited to the study, of whom 219 (3%) children were excluded: 181 did not meet the eligibility criteria, 32 did not have baseline data and six were withdrawn. Of the 8394 children recruited and retained, clinicians recorded their gut feeling for 8377 (99.8%) - all analyses that follow use these 8377 children. Participant flow through the study is available in Fig. 2. Oxygen saturation was removed from all analysis as these data were missing for 50% of children due to a lack of available paediatric oxygen saturation monitors. All other reported variables had < 2% missing data. Children’s median age was 3 years (range 3 months-15 years, IQR, 1-6 years) and 1390 (17%) were under 1 year. There were slightly more boys (52%) than girls, and of the 8333 (99.5%) for whom ethnicity data were available the majority (6540, 78%) were white (similar to UK Census 2011 data) [29]. Families’ median deprivation (IMD) score was 16.7 (IQR 8.8-29.5), similar to the English median (17.2, IQR 9.8-30.2) [20]. Median clinician reported illness severity was 3 (range 0-9, IQR 2-4).

Clinicians reported a gut feeling something was wrong in 1706 (20%) cases. 3110 (37%) were prescribed an antibiotic, with 2341 (28%) given an immediate script and 769 (9%) given a delayed script. Seventy-four children were immediately referred for secondary care assessment at the recruiting consultation, of whom 13 were subsequently admitted. A further 65 children were admitted to hospital with a RTI in the subsequent 30 days. For the 78 hospitalised children the median number of days to hospitalisation was 2 days (IQR 1-12 days). The hospital discharge diagnoses were: lower respiratory tract infection (19%); bronchiolitis (18%); viral wheeze (15%); upper respiratory tract infection (13%); croup (8%); infected exacerbation of asthma (8%); tonsillitis (6%); viral illness (5%); febrile illness (3%); and pneumonia (1%). Of the 8193 children for whom data were available relating to primary care reconsultations in the 30 days following baseline, 1847 reconsulted for an RTI. Of these 354 (4%) reconsulted for the same illness with evidence of symptom deterioration, with a median of 5 days (IQR 2-11 days) to this reconsultation. The percentage inter-rater agreement (kappa) for hospitalisation and reconsultation for the same illness with evidence of deterioration were 90% (Kappa 0.80) and 84% (Kappa 0.67) respectively. There were 242/8377 (3%) children in whom the clinician reported a low illness severity score (≤2) whilst also reporting they a gut feeling something was wrong.

### Determinants of gut feeling

#### Univariable analyses

Fifty-one (84%) of the 61 variables measured were associated with a gut feeling (with *p*-values < 0.05 (Additional file 1: Web Appendix, Table S1). Two of the three clinician characteristics were associated: those with more years since qualification and NPs reported having a gut feeling in a higher proportion of the children than GPs (27% vs. 18%), while additional qualifications were not associated. Only one of the five sociodemographic variables collected were associated: being male (55% vs 51%). There was no evidence of a difference in the children’s ethnicity, age, the mother’s age at the time of the child’s birth, or home IMD score in the two groups. All the four variables collected regarding the child’s medical history were associated with gut feeling. The child was more likely to: have a chronic condition (21% vs 18%), a previous or current asthma diagnosis (12% vs 8%), and to have consulted for an RTI on two or more occasions in the 12 months prior to recruitment (36% vs 35%), than the children for whom the clinician did not have a gut feeling. There was evidence that 44/49 (90%) of the symptoms and signs that were recorded at baseline were associated with gut feeling. The five symptoms and signs that did not reach the 5% threshold were low illness duration prior to recruitment, barking or croupy cough, blocked or runny nose, diarrhoea and stridor (Additional file 1: Web Appendix, Table S1).

#### Multivariable analysis

There was strong evidence that increasing years since clinician qualification, five carer-reported symptoms and eight clinically-reported signs were independently associated with gut feeling (Table 1). The strongest was crackles and crepitations. In the multivariable model there was no evidence of association with clinician type (NP versus GP), additional clinician qualifications or any of the socio-demographic variables. There was strong evidence that clustering by clinician was important in the model (LRT of rho = 0: *p* < 0.001) when compared with the multivariable model without clustering. The AUROC curve was 0.82, 95% confidence interval (CI) 0.80-0.83. Imputing the missing data for the final model made little difference to the odds ratios, confidence intervals and *p*-values. The sensitivity analysis exploring the impact of high clinician illness score on the model resulted in few changes: high clinician illness severity score was retained, while severe fever in the last 24 h and parent reporting that that illness was worse recently dropped out of the model. There was little change to the remaining estimates, except for crackles and crepitations, where the OR fell from 14.56 (95% CI 11.71-18.12) to 9.57 (95% CI 7.64-11.99) and the AUROC increased from 0.81 (95% CI 0.80-0.83) to 0.82 (95% CI 0.83-0.85).

**Table 1:** Determinants: Multivariable associations between parent-reported symptoms, clinician-reported observations, clinician profile and ‘gut feeling’

Significant predictors	No gut feeling		Yes gut feeling		OR	95% CI	*p*-value
	n/N	%	n/N	%			
Years qualified (Categorical)							
1-9 years	646/720	89.7	74/720	10.3	Ref	Ref	< 0.001 5 d.f.^*^
10-14 years	1790/2213	80.9	423/2213	19.1	0.55	0.30-1.02	
15-19 years	971/1206	80.5	235/1206	19.5	1.44	0.71-2.90	
20-24 years	1219/1586	76.9	367/1586	23.1	1.28	0.57-2.87	
25-29 years	875/1159	75.5	284/1159	24.5	1.67	0.76-3.68	
30+ years	1170/1493	78.4	323/1493	21.6	1.08	0.47-2.46	
Parent reported symptoms							
High parent illness severity score (≥7/10)	1459/6650	21.9	681/1702	40.0	1.67	1.39-2.01	< 0.001
Fever in the last 24 h (severe)	363/6647	5.5	177/1700	10.4	1.66	1.23-2.26	0.001
Low energy during illness	3404/6669	51.0	1100/1704	65.6	1.65	1.36-1.99	< 0.001
Shortness of breath during illness	2115/6670	31.7	857/1704	50.3	1.50	1.25-1.81	< 0.001
Illness worse recently	4182/6663	62.8	1339/1704	78.6	1.44	1.18-1.76	< 0.001
Clinical signs							
Crackles and crepitations	695/6661	10.4	901/1704	52.9	14.56	11.71-18.12	< 0.001
Recession	164/6663	2.5	239/1704	14.0	3.31	2.29-4.79	< 0.001
Pallor	6185/6663	7.2	345/1705	20.2	3.10	2.30-4.18	< 0.001
Bronchial breath	136/6658	2.0	142/1703	8.3	2.99	1.90-4.70	< 0.001
Wheeze (recorded in consultation)	675/6663	10.1	560/1703	32.9	2.78	2.23-3.46	< 0.001
High temperature (≥37.8 °C recorded in consultation)	629/6653	9.5	415/1702	24.4	2.32	1.84-2.91	< 0.001
High respiratory rate (age related cut offs)	825/6634	12.4	414/1698	24.4	1.64	1.30-2.07	< 0.001
Inflamed pharynx	1873/6647	28.2	521/1702	30.6	1.51	1.23-1.86	< 0.001

*N* = 8217/8377 (98.1% of the sample) 514 clusters (clinicians)

^*^Degrees of freedom

### Management decisions and gut feeling

There was strong evidence in both the univariable and multivariable analysis that when clinicians report a gut feeling, they were more likely to prescribe antibiotics and refer for same day further secondary care assessment (Table 2, including description of the covariates retained in the multivariate model at the < 1% level). These findings were independent of clinician’s impression of illness severity.

**Table 2:** Management decisions: relationship between ‘gut feeling’ and subsequent clinical management

Treatment	No gut feeling		Yes gut feeling		Univariable analysis controlling for clustering			Multivariable analysis controlling for clustering and significant covariates		
	n/N	%	n/N	%	OR	95% CI	*P*-value	OR	95% CI	*P*-value
Prescribed antibiotics at baseline consultation										
Any antibiotics prescribed	1707/6671	25.6	1403/1706	82.2	20.80	17.42-24.83	< 0.001	5.85	4.67-7.32	< 0.001^±^
Referred for acute admission (during the recruiting consultation)										
Referral for acute admission	16/6671	0.2	58/1706	3.4	19.27	9.83-37.79	< 0.001	12.64	6.31-25.32	< 0.001^¥^

^±^Co-variates of any antibiotic prescription (retained at the < 1% level): illness has got worse recently, child’s age (< 2 years), barking cough, fever, diarrhoea, low energy and productive cough during the illness, moderate/severe wheeze and severe fever in the last 24 h, recession, crackles and crepitations, wheeze (as reported by the clinician), bronchial breath, inflamed pharynx, high temperature, high clinician and parent illness severity scores, low illness duration and gut feeling that something is wrong

^¥^Co-variates of referral for acute admission (retained at the < 1% level): Recession, gut feeling that something is wrong and capillary refill time (CRT)

### Prognostic value of gut feeling

There was no evidence to suggest that the children for whom the clinician had a gut feeling were more likely to re-consult for the same illness with evidence of illness deterioration in the 30 days following recruitment in the univariable or multivariable analysis. They were over twice as likely to be admitted to hospital for a RTI in the 30 days following recruitment. However, after adjusting for other variables associated with hospital admission, this relationship was attenuated. This was also true for the sensitivity analysis examining the 7-day hospitalisation outcome. The presence of clinician reported wheeze and/or recession in the multivariate model reduced the association with gut feeling below the < 5% threshold (Table 3, including description of covariates retained in the multivariate model). There was evidence of clinician clustering effect for re-consultation (LRT of rho = 0: *p* < 0.001) but not hospitalisation (30 days LRT of rho = 0: *p* = 0.30, 7 days LRT of rho = 0: *p* = 0.43), indicating that there was a relationship between the clinician who had seen the child and the likelihood of child reconsulting, but not being hospitalised. There was also no evidence of an association between hospitalisation and the more focused definition of gut feeling (described in previous studies) was explored at univariate level(OR 1.26, 95% CI 0.38-4.13, *p* = 0.71).

**Table 3:** Prognosis: relationship between ‘gut feeling’ and children’s health outcomes

Health outcome	No gut feeling		Yes gut feeling		Univariable analysis controlling for clustering			Multivariable analysis controlling for clustering and significant covariates		
	n/N	%	n/N	%	OR	95% CI	*P*-value	OR	95% CI	*P*-value
Health outcome										
Re-consultation for the same illness with evidence of illness deterioration (≥1) in the 30 days following recruitment	271/6523	4.2	83/1670	5.0	1.30	0.99 -1.71	0.06	1.08	0.80-1.46	0.60^¥^
Admitted to hospital in the 30 days following recruitment	48/6671	0.7	30/1706	1.8	2.47	1.55-3.95	< 0.001	1.11	0.65-1.89	0.71^≠^
Sensitivity analysis										
Admitted to hospital in the ≤7 days post recruitment	24/6647	0.4	25/1681	1.5	4.20	2.34-7.54	< 0.001	1.62	0.83-3.14	0.16^α^

^¥^Co-variates of re-consultation for the same RTI with evidence of deterioration (retained at the < 5% level): ≥2 consultations for RTI in primary care in the year prior to baseline, low illness duration prior to baseline, previous asthma diagnosis, current asthma diagnosis, child’s age at baseline (< 2 years), white ethnicity, parent reported barking cough, wheeze and taking fewer fluids during the illness, moderate/severe vomiting and fewer fluids in the 24 h prior to baseline, severe eating less in the 24 h prior to baseline, wheeze and recession (as reported by the clinician)

^≠^Co-variates of hospitalisation (retained at the < 5% level): child’s age (< 2 years), low illness duration prior to baseline, current asthma diagnosis, moderate/severe vomiting, recession, wheeze (as reported by the clinician), high temperature (age related cut offs)

^α^Co-variates of hospitalisation ≤7 days post recruitment (retained at the < 5% level): child’s age (< 2 years), low illness (< 3 days) duration prior to baseline, current asthma diagnoses, moderate/severe vomiting, recession, wheeze (as reported by the clinician), high temperature (age related cut offs)

## Discussion

### Summary of findings

In a large, rigorously conducted, prospective cohort study, gut feeling was more commonly reported by more experienced clinicians and in children with parent-reported symptoms and physical examination signs suggesting more severe illnesses. Gut feeling was strongly associated with management decisions – both antibiotic prescribing and same-day referral for admission. Despite this, we did not find strong evidence that gut feeling was associated with indicators of poorer prognosis in the following 30 days, such as reconsulting in primary care with evidence of illness deterioration or being hospitalised for a RTI, at least not after controlling for potential confounders.

### Strengths and limitations

To our knowledge, ours is the largest, prospective study to describe the determinants, management decisions and prognostic value of gut feeling for respiratory tract infections in children in routine, office-based primary care [2]. We included a broad range of clinician, demographic, child and clinical characteristics and the study focussed on a clinical group in whom clinicians report diagnostic and prognostic uncertainty [6]. Determinants of gut feeling were measured according to routine clinical practice using standardised reporting forms on the day of recruitment, blind to the prognostic outcomes, which were reliably measured from the primary care record. We used rigorous techniques to adjust for confounding.

We are aware of five main limitations. First, while we offered some instructions to support completion of the question “My gut feeling is ‘something is wrong’”, including a definition, there was no attempt to interpret it and clinicians were simply asked to report its presence/absence. While the low question refusal suggests clinicians understood the question, it is likely the presence of gut feeling means different things to different clinicians. Definitions of gut feeling overlap, and may not be clear to clinicians. Given our relatively high (20%) prevalence of gut feeling, the strong associations found with symptoms and signs (such as crepitations/crackles) and with high clinician reported illness severity score, it is likely our clinicians were using a broad definition, such as “the presence of a significant illness”. Second, although focussing on an extremely common primary care presentation, [2] findings may not generalise to other illnesses or different age groups. Third, we adjusted for a wide range of potential confounders but it is possible that other unmeasured factors could be important. We are aware of another study that found ‘parent concern that the illness was different to other illnesses’, weight loss and history of convulsions have been shown to be associated with gut feeling and these were not collected in our study [15]. Fourth, while our conservative model entry and exit thresholds have minimised scope for Type I error, the relative infrequency of the hospitalisation outcome, and the fact this was a secondary analysis of data collected for another purpose, [18] could have led to Type II error and us incorrectly concluding that gut feeling does not have prognostic value. The large study size mitigates against this for re-consultation. It is plausible some cases of re-consultation or hospitalisation were avoided where antibiotics were given and this may mask associations with gut feeling (so-called confounding by indication). However, there is limited evidence of the mitigating effects of antibiotics on prognostic outcome for RTI. Two studies, one with adults [30] and the other with children (in submission [31]) with RTIs found antibiotics did not reduce hospitalisation, and immediate antibiotics did not reduce reconsultations. Finally, the outcomes used to explore the prognostic value of gut feeling are indicators of poor health, but we acknowledge that other factors beyond the child’s illness severity can influence these, such as parent behaviour and available services in the area.

### Comparison with existing literature

#### Determinants of gut feeling

Previous studies have described gut feeling in primary care clinicians as being derived from a combination of patient characteristics and clinicians’ knowledge and past experience [32, 33]. Like others we found that pallor or appearance, energy and breathing patterns were associated with gut feeling [5, 15].

Our results contrast with a previous study, which did not find evidence to support their hypothesis that high temperature contributed towards gut feeling, but we found high temperature and carer-reported severe fever both contributed [15]. The literature is contradictory regarding the role of clinician experience. For instance, both we and a previous qualitative study found more experienced family primary physicians were more likely to report having a gut feeling something was wrong, [17] whereas another quantitative study found that more senior doctors were less likely to report it [15]. These differences may be due to differences in participating children or definition of gut feeling; van den Bruel’s study included children with any non-serious acute illness, whereas we studied children with RTI regardless of clinician-reported illness severity. In their study they conceptualised gut feeling as “intuitive feeling that something was wrong even if the clinician was unsure why” and included the 3.1% children where the clinician reported a gut feeling in addition to the illness was not severe. Our definition was broader and included 20% of the children. However, in our sample a clinician reported a gut feeling combined with a low illness severity score approximates this definition, and we found the same proportion (3%). The concept that gut feeling is more commonly reported in more experienced clinicians is supported by the learning perspective of intuition: where intuition is the consequence of unconscious drawing on an implicit knowledge base and recognition of patterns of clinical markers that is developed though experience and may be associated with previous negative or uncertain outcomes. As demonstrated by our findings, more experienced does not mean more accurate. The validity of the gut feeling as a guide to decision-making relies not only on the quantity but also the quality of the feedback. If there is good feedback during the learning process, this results in good or accurate gut feeling and associated decision-making. Conversely, poor or absent feedback results in poor intuition [34].

#### Management decisions and gut feeling

Our findings agree with previous studies [32] that found gut feeling can play a substantial role in treatment decisions.

#### Prognostic value of gut feeling

A recent systematic review suggested that gut feeling was of greater diagnostic value than the majority of symptoms and signs [17]. Two recent prospective studies reported that gut feeling in primary care was also associated with serious infectious illness in children in Belgium [15] and serious disease in adults and children in Denmark [35]. In contrast, we did not find evidence that gut feeling was associated with subsequent reconsultations with evidence of deterioration. When considered in isolation, we found gut feeling was associated with hospitalisation, however, when other clinical signs (wheeze and/or recession) were taken into account, gut feeling was no longer associated. Indeed, our recent paper identified seven other characteristics that were independently associated with hospitalisation – namely age less than 2 years, current asthma, illness duration of 3 days or less, parent-reported moderate or severe vomiting in the previous 24 h, severe fever in the previous 24 h or a body temperature of 37·8 °C or more at presentation, clinician-reported intercostal or subcostal recession, and wheeze on auscultation [3]. Use of these may reduce prognostic uncertainty and reduce reliance on gut feeling.

### Clinical, teaching and research implications

Our study suggests that for children presenting to primary care with acute cough and RTI, the gut feeling that something is wrong, which appears to drive clinical decision-making in this group, are not associated with either re-consultation or hospital admission, at least not after potential confounding effects were taken into account [30, 31]. Given the high levels of uncertainty reported by clinicians looking after this group of patients, [5, 36] it is not surprising they wish to use all the tools at their disposal, including gut feeling. However, our results suggest some clinicians may over-value gut feeling in their clinical decision making in this group of patients and that they are using gut feeling as a proxy or unconscious marker of other important symptoms and signs. Gut feeling may have value in the absence of known, objectively assessed markers of risk, however, attending to objective assessment of these risks is preferable when this information is available. Future research may wish to focus on a more specific definition of gut feeling, including perhaps the sense of dissonance arising when conscious and unconscious assessment processes are in conflict.

## Conclusions

Clinicians were more likely to report a gut feeling something is wrong, when they were more experienced and when children were more unwell. Gut feeling is independently and strongly associated with antibiotic prescribing and referral to secondary care, but not with two indicators of poor child health.

## Additional file

Additional file 1: Web Appendix Table S1. Determinants: univariable associations between sociodemographic, parent-reported symptoms, clinician-reported observations, clinician profile and gut feeling. This table presents the univariable associations between child and clinician variables and gut feeling. (DOCX 21 kb)

## Abbreviations

AUROC: Area under the receiver operating Characteristic
CRF: Case report form
CRT: Capillary refill time
DCH: Diploma in child health
GPs: General practitioners
IMD: Indices of multiple deprivation
IQR: Inter-quartile range
LRT: Likelihood-ratio test
MRCGP: Membership of the royal college of general Practitioners
NPs: ‘Prescribing’ nurse practitioners
RTI: Respiratory tract infection
STROBE: Strengthening the reporting of observational studies in epidemiology
