# Supplementary material for: What gives rise to clinician gut feeling, its influence on management decisions and its prognostic value for children with RTI in primary care: a prospective cohort study
Source: BMC Fam Pract. 2018 Feb 5;19:25. doi: 10.1186/s12875-018-0716-7 (PMC5800050; doi:10.1186/s12875-018-0716-7)
Supplement: Additional file 1: — Web Appendix Table S1. Determinants: univariable associations between sociodemographic, parent-reported symptoms, clinician-reported observations, clinician profile and gut feeling. This table presents the univariable associations between child and clinician variables and gut feeling. (DOCX 21 kb) [file 12875_2018_716_MOESM1_ESM.docx]

Web Appendix

Table 4**. Determinants: univariable associations between sociodemographic, parent-reported symptoms, clinician-reported observations, clinician profile and gut feeling**

| **Predictor** | **No gut feeling** | | **Yes gut feeling** | | **OR** | **95% CI** | **P-value** |
| --- | --- | --- | --- | --- | --- | --- | --- |
| **SOCIO-DEMOGRAPHIC VARIABLES** | | | | | | | |
|  | **Median** | **IQR** | **Median** | **IQR** |  |  |  |
| Age (years) | 3 | 1-6 | 3 | 1-6 | 1.00 | 0.95-1.02 | 0.86 |
|  | **n/N** | **%** | **n/N** | **%** |  | | |
| Gender (male) | 3383/6671 | 50.7% | 940/1706 | 55.1% | 1.21 | 1.07-1.38 | 0.003 |
| Young mother (≤ 26 years) | 1948/6652 | 29.3% | 482/1699 | 28.4% | 0.87 | 0.75-1.01 | 0.06 |
| IMD score (high, top quintile) | 132/6512 | 20.3% | 312/1672 | 18.7% | 0.85 | 0.70-1.04 | 0.11 |
| Ethnicity (white) | 5123/6638 | 77.2% | 1417/1695 | 83.6% | 1.01 | 0.83-1.23 | 0.94 |
| **PAST MEDICAL HISTORY** | | | | | | | |
| Consultations for RTI in the 12 months prior to baseline (≥2) | 2258/6556 | 34.6% | 608/1672 | 36.4% | 1.29 | 1.13-1.48 | <0.001 |
| Asthma (current diagnosis) | 543/6671 | 8.1% | 205/1706 | 12.0% | 1.44 | 1.17-1.78 | 0.001 |
| Asthma (previous diagnosis) | 255/6670 | 3.8% | 98/1706 | 5.7% | 1.41 | 1.05-1.89 | 0.022 |
| Chronic conditions (any) | 1213/6670 | 18.2% | 364/1704 | 21.4% | 1.32 | 1.13-1.55 | 0.001 |
| **PARENT-REPORTED SYMPTOMS (PRESENT DURING THE ILLNESS)** | | | | | | | |
| Low illness duration prior to recruitment (≤3 days) | 1913/6668 | 28.7% | 474/1706 | 27.8% | 1.02 | 0.88-1.17 | 0.81 |
| Breathing faster than normal | 2115/6670 | 31.7% | 857/1704 | 50.3% | 3.36 | 2.92-3.88 | <0.001 |
| High parent illness severity score (≥7) | 1459/6650 | 21.9% | 681/1702 | 40.0% | 3.11 | 2.70-3.58 | <0.001 |
| Low energy/fatigue/lethargy | 3404/6669 | 51.0% | 1100/1704 | 64.6% | 2.67 | 2.31-3.09 | <0.001 |
| Fever | 3932/6669 | 59.0% | 1235/1705 | 72.4% | 2.58 | 2.23-2.98 | <0.001 |
| Eating less | 3838/6667 | 57.7% | 1180/1704 | 69.3% | 2.51 | 2.17-2.89 | <0.001 |
| Illness much worse recently | 4182/6663 | 62.8% | 1339/1704 | 78.6% | 2.38 | 2.03-2.79 | <0.001 |
| Disturbed sleep | 5018/6668 | 75.3% | 1427/1704 | 83.7% | 2.14 | 1.80-2.55 | <0.001 |
| Wheezing or whistling in the chest | 2454/6668 | 36.8% | 823/1704 | 48.3% | 2.07 | 1.81-2.37 | <0.001 |
| Chills/Shivering | 1366/6669 | 20.5% | 490/1704 | 28.8% | 1.90 | 1.64-2.20 | <0.001 |
| Taken fewer fluids/milk feeds | 1986/6665 | 29.8% | 656/1705 | 38.5% | 1.85 | 1.62-2.12 | <0.001 |
| Productive wet cough | 3440/6665 | 51.6% | 1047/1705 | 61.4% | 1.69 | 1.47-1.94 | <0.001 |
| Vomiting (including after a cough) | 1802/6669 | 27.0% | 540/1705 | 31.7% | 1.66 | 1.45-1.92 | <0.001 |
| Passing urine less often/dryer nappies | 857/6657 | 12.9% | 280/1702 | 16.5% | 1.65 | 1.37-1.98 | <0.001 |
| Change in cry | 1074/6657 | 16.1% | 306/1692 | 18.1% | 1.56 | 1.31-1.86 | <0.001 |
| Dry cough | 4109/6669 | 61.6% | 900/1703 | 52.9% | 0.64 | 0.56-0.73 | <0.001 |
| Diarrhoea | 966/6669 | 14.5% | 267/1705 | 15.7% | 1.20 | 1.00-1.43 | 0.047 |
| Barking/croupy cough | 1692/6666 | 25.4% | 448/1703 | 26.3% | 1.04 | 0.89-1.21 | 0.61 |
| Blocked/runny nose | 5386/6669 | 80.8% | 1311/1705 | 76.9% | 0.99 | 0.85-1.17 | 0.95 |
| **PARENT-REPORTED SYMPTOMS (LAST 24 HOURS)** | | | | | | | |
| Change in cry (mod/severe) | 612/6648 | 9.2% | 174/1691 | 10.3% | 1.54 | 1.24-1.93 | <0.001 |
| Vomiting (mod/severe) | 634/6661 | 9.5% | 204/1704 | 12.0% | 1.77 | 1.44-2.16 | <0.001 |
| Disturbed sleep (severe) | 1025/6638 | 15.4% | 325/1698 | 19.1% | 1.78 | 1.48-2.13 | <0.001 |
| Taking fewer fluids/milk feeds (mod/severe) | 864/6656 | 13.0% | 291/1697 | 17.2% | 1.92 | 1.60-2.29 | <0.001 |
| Passing urine less often/dryer nappies (mod/severe) | 330/6647 | 5.0% | 132/1701 | 7.8% | 1.97 | 1.51-2.57 | <0.001 |
| Productive wet cough (severe) | 488/6650 | 7.3% | 189/1700 | 11.1% | 2.08 | 1.67-2.59 | <0.001 |
| Chills/Shivering (mod/severe) | 579/6663 | 8.7% | 256/1700 | 15.1% | 2.17 | 1.78-2.64 | <0.001 |
| Eating less (severe) | 301/6645 | 4.5% | 128/1695 | 7.6% | 2.34 | 1.79-3.07 | <0.001 |
| Low energy/fatigue/lethargy (mod/severe) | 1654/6646 | 24.9% | 616/1695 | 36.3% | 2.47 | 2.12-2.87 | <0.001 |
| Wheeze (mod/severe) | 1135/6659 | 17.0% | 478/1703 | 28.1% | 2.67 | 2.28-3.13 | <0.001 |
| Fever (severe) | 363/6647 | 5.5% | 177/1700 | 10.4% | 3.02 | 2.39-3.82 | <0.001 |
| Breathing faster than normal (mod/severe) | 1102/6662 | 16.5% | 525/1700 | 30.9% | 3.70 | 3.15-4.34 | <0.001 |
| Blocked/runny nose (severe) | 517/6634 | 7.8% | 153/1698 | 9.0% | 1.50 | 1.19-1.89 | <0.001 |
| Dry cough (severe) | 437/6647 | 6.6% | 124/1701 | 7.3% | 1.42 | 1.10-1.84 | 0.008 |
| Barking/croupy cough (mod/severe) | 1194/6661 | 17.9% | 335/1699 | 19.7% | 1.21 | 1.02-1.44 | 0.03 |
| Diarrhoea | 276/6665 | 4.1% | 68/1704 | 4.0% | 0.94 | 0.48-1.85 | 0.87 |
| **CLINICAL SIGNS** | | | | | | | |
| Inter/subcostal recession | 164/6663 | 2.5% | 239/1704 | 14.0% | 13.14 | 9.87-17.49 | <0.001 |
| Bronchial Breathing (Unilateral/ Bilateral) | 136/6658 | 2.0% | 142/1703 | 8.3% | 9.44 | 6.68-13.3 | <0.001 |
| Nasal Flaring | 47/6664 | 0.7% | 55/1705 | 3.2% | 9.39 | 5.68-15.52 | <0.001 |
| Pallor | 478/6663 | 7.2% | 345/1705 | 20.2% | 5.53 | 4.39-6.97 | <0.001 |
| Wheeze (Unilateral/Bilateral) | 675/6663 | 10.1% | 560/1703 | 32.9% | 5.24 | 4.44-6.18 | <0.001 |
| Abnormal consciousness | 70/6660 | 1.1% | 58/1703 | 3.4% | 4.31 | 2.84-6.54 | <0.001 |
| High temp ≥37.8°C | 629/6653 | 9.5% | 415/1702 | 24.4% | 4.30 | 3.61-5.12 | <0.001 |
| High respiratory rate (age related cut offs) | 825/6634 | 12.4% | 414/1698 | 24.4% | 3.40 | 2.84-4.06 | <0.001 |
| High pulse (age related cut off) | 256/6629 | 3.9% | 134/1695 | 7.9% | 3.05 | 2.34-3.99 | <0.001 |
| Inflamed pharynx | 1873/6647 | 28.2% | 521/1702 | 30.6% | 1.85 | 1.58-2.16 | <0.001 |
| Grunting | 33/6662 | 0.5% | 42/1705 | 2.5% | 7.80 | 4.42-13.76 | <0.001 |
| Crackles/crepitations (Unilateral/Bilateral) | 695/6661 | 10.4% | 901/1704 | 52.9% | 25.37 | 20.82-30.91 | <0.001 |
| Slow capillary refill time (≥3 seconds) | 51/6645 | 0.8% | 18/1695 | 1.1% | 1.97 | 1.02-3.83 | 0.045 |
| Stridor | 30/6661 | 0.5% | 14/1705 | 0.8% | 1.85 | 0.84-4.09 | 0.13 |
| **CLINICIAN-RELATED VARIABLES** | | | | | | | |
| Clinician type (GP vs. nurse) | 5139/6587 | 78.0% | 1129/1654 | 68.3% | 0.53 | 0.33-0.87 | 0.01 |
| Any additional qualification | 945/1513 | 62.5% | 189/299 | 63.2% | 0.70 | 0.34-1.41 | 0.32 |
| No. of years qualified at time of recruitment  1-9 years  10-14 years  15-19 years  20-24 years  25-29 years  30+ years | 646/720  1790/2213  971/1206  1219/1586  875/1159  1170/1355 | 89.7%  80.9%  80.5%  76.9%  75.5%  80.0% | 74/720  423/2213  235/1206  367/1586  284/1159  323/1355 | 10.3%  19.1%  19.5%  23.1%  24.5%  20.0% | Ref  1.39  2.30  1.99  2.72  2.29 | Ref  0.90-2.16  1.38-3.83  1.10-3.58  1.53-4.84  1.25-4.18 | 0.002 |
